# Model eggs fail to detect egg recognition in host populations after brood parasitism is relaxed

**DOI:** 10.1186/s12983-020-00362-0

**Published:** 2020-05-12

**Authors:** Canchao Yang, Longwu Wang, Shun-Jen Cheng, Yu-Cheng Hsu, Anders Pape Møller, Wei Liang

**Affiliations:** 1 Ministry of Education Key Laboratory for Ecology of Tropical Islands, College of Life Sciences, Hainan Normal University, Haikou, 571158 China; 2 State Forestry Administration of China Key Laboratory for Biodiversity Conservation in Mountainous Areas of Southwest Karst, School of Life Sciences, Guizhou Normal University, Guiyang, 550001 China; 3 Department of Natural Resources and Environmental Studies, National Dong Hwa University, 97401 Hualien, Taiwan; 4 Ecologie Systématique Evolution, Université Paris-Sud, CNRS, AgroParisTech, Université Paris-Saclay, F-91405 Orsay Cedex, France

**Keywords:** Avian brood parasitism, *Cuculus canorus*, Parasitism pressure, *Prinia inornata*, Rejection motivation

## Abstract

**Background:**

Obligate brood parasites exert strong selective pressure on target hosts. In response, hosts typically evolve anti-parasitism strategies, of which egg recognition is one of the most efficient. Generally, host egg-recognition capacity is determined using model eggs. Previous studies have shown that some host species, which are capable of detecting parasite eggs, do not reject model eggs. However, it is unknown that whether the reaction to model eggs varies among distinct populations of the same host in relation to the degree of parasitism pressure.

**Results:**

Here, we compared the rejection frequencies of model eggs and real eggs between mainland and island populations of the plain prinia (*Prinia inornata*), which are respectively sympatric and allopatric with their brood parasite, the common cuckoo (*Cuculus canorus*). Our results indicated that the mainland and island populations rejected real eggs at similar rates, but rejected model eggs, which were similar in size to real eggs but heavier, at significantly different rates: the island population rejected fewer model eggs, possibly because the rejection motivation of this population was lower due to absence of parasitism.

**Conclusions:**

Our results indicated that some factors affecting the decision to reject, such as rejection motivation, varied according to the degree of parasitism pressure, and thus influenced the frequency of egg rejection. Furthermore, our results suggested that model eggs should be used with caution in comparative studies of egg recognition abilities among species or populations subjected to different intensities of brood parasitism. That is, model eggs may fail to accurately detect egg recognition in host populations with little to no risk of parasitism.

## Background

Obligate brood parasites are birds that do not build nests, incubate eggs, or rear their own offspring; instead, these birds transfer such duties to other bird species, which are used as hosts [1, 2]. As the host is forced to provide parental care and to raise unrelated young, host fitness is dramatically reduced or entirely eliminated [3]. Natural selection thus favors hosts that have evolved anti-parasite adaptations, of which egg recognition is one of the most general and efficient [2]. At present, egg recognition by brood-parasite hosts is typically assayed using model eggs, which are generally made of polymer clays; egg recognition behaviors are generally considered confirmed if the model egg is rejected by the host [1, 4, 5]. However, unlike real eggs, model eggs are solid and hard. Model eggs are therefore difficult for small host species to reject, as the bills of these hosts are too small to grasp the model egg; such species must reject model eggs using puncturing behaviors [6, 7]. Indeed, even grasp-ejector hosts may accept model eggs that have been successfully recognized as foreign, implying that successful recognition does not always result in successful rejection [6, 7]. Therefore, artificial parasitism experiments using model eggs may not always accurately detect the egg recognition capacities of hosts. Other factors, such as rejection motivation, may influence the host decision to reject an egg [7, 8]. Although model eggs have been shown to be unsuitable for tests of egg recognition in small bill hosts [9], and heavier model eggs are generally accepted more frequently [10], it remains unclear whether distinct populations of the same host, subject to varying degrees of egg parasitism pressure, react differently to model eggs. That is, non-parasitised host populations may be more likely to accept difficult-to-reject model eggs in comparison to heavily-parasitised host populations, because hosts with lower risks of parasitism have higher motivation thresholds [8].

To determine whether model eggs induced similar reactions among host populations subject to different degrees of parasitism pressure, we compared egg rejection behaviors between mainland and island populations of the plain prinia (*Prinia inornata*), using both real and model eggs. We used model eggs that were similar in size to host eggs, but heavier. The mainland population of the plain prinia is parasitised by the common cuckoo (*Cuculus canorus*) [11, 12]. In contrast, the island population is not parasitised by the common cuckoo; no common cuckoos live on the island, which is 200 km from the mainland [13]. The aim of this study was to determine whether the use of model eggs had different effects on the rejection behaviors of these host populations. Real eggs were expected to be more easily ejected by the hosts than the model eggs. Thus, if model eggs have the same effect on rejection behaviors, irrespective of parasitism pressure, we would expect that both populations would reject model and real eggs at the same frequency. Alternatively, if parasitism pressure affects the likelihood of model egg rejection, we would expect that the frequencies of real and model egg rejection would differ between populations.

## Materials and methods

### Study area and study species

This study was performed on mainland China and Taiwan Island in April and July, 2012 and 2013. The experiments on the mainland population of plain prinia were performed in Nonggang National Nature Reserve, Guangxi, Central China (23° 39′ N, 107° 04′ E). Although the egg morph of the cuckoo parasitizing the plain prinias was unknown, the plain prinia has been identified as one of the major hosts of common cuckoo [14]. In the studied location, many species of parasitic cuckoos, including the common cuckoo, coexist and breed sympatrically with the plain prinia [12]. The plain prinia population studied in Taiwan was located in Shoufeng, Hualien County (23° 51′ N, 121° 31′ E). Taiwan Island, which is southeast of mainland China, has been geographically separated from the mainland for 2–3 million years [15, 16]. The common cuckoo does not breed on Taiwan Island [12]. That is, the mainland population is sympatric with, and under parasitism pressure from, the common cuckoo, while the Taiwanese population is not parasitized. Therefore, these populations of the plain prinia provide an excellent opportunity to identify differences in the effects of model eggs on egg rejection behaviors between populations subjected to dissimilar intensities of brood parasitism.

### Parasitic eggs

**Fig. 1:**
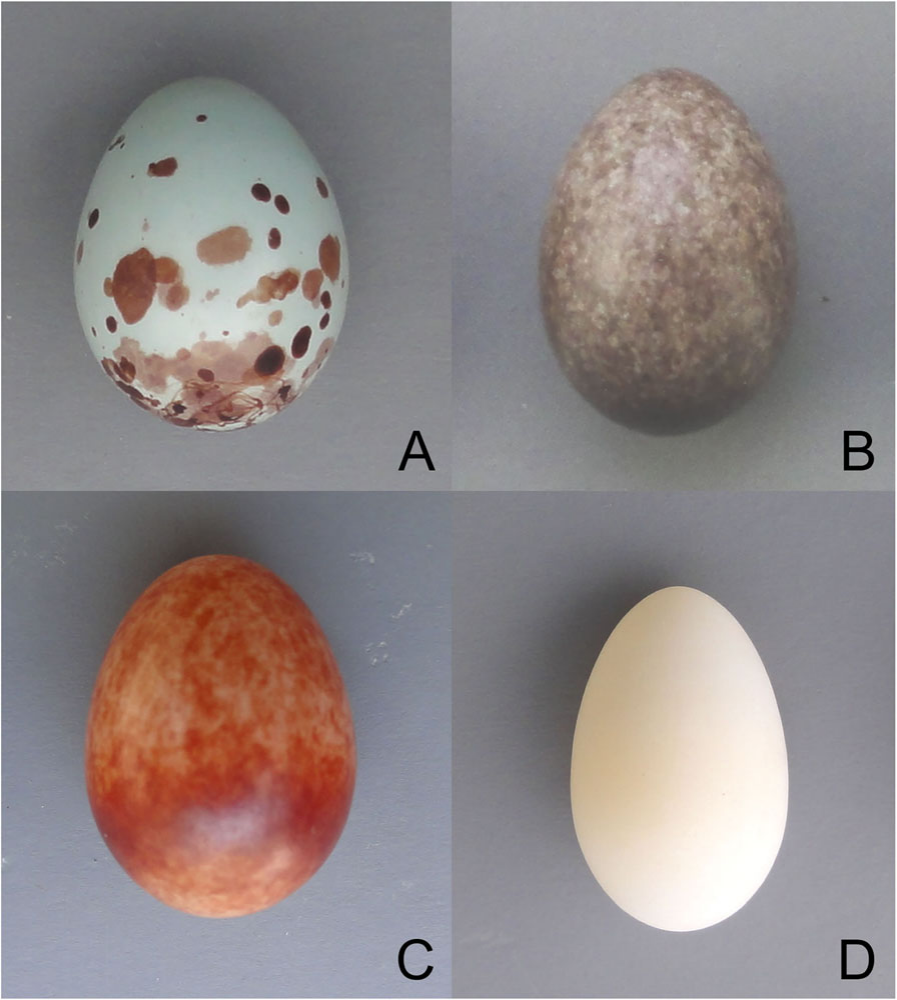
Representative real eggs used for the parasitism experiments. **a** plain prinia (*Prinia inornata*), **b** tree sparrow (*Passer montanus*), **c** yellow-bellied prinia (*Prinia flaviventris*), and **d** white-rumped munia (*Lonchura striata*)

**Fig. 2:**
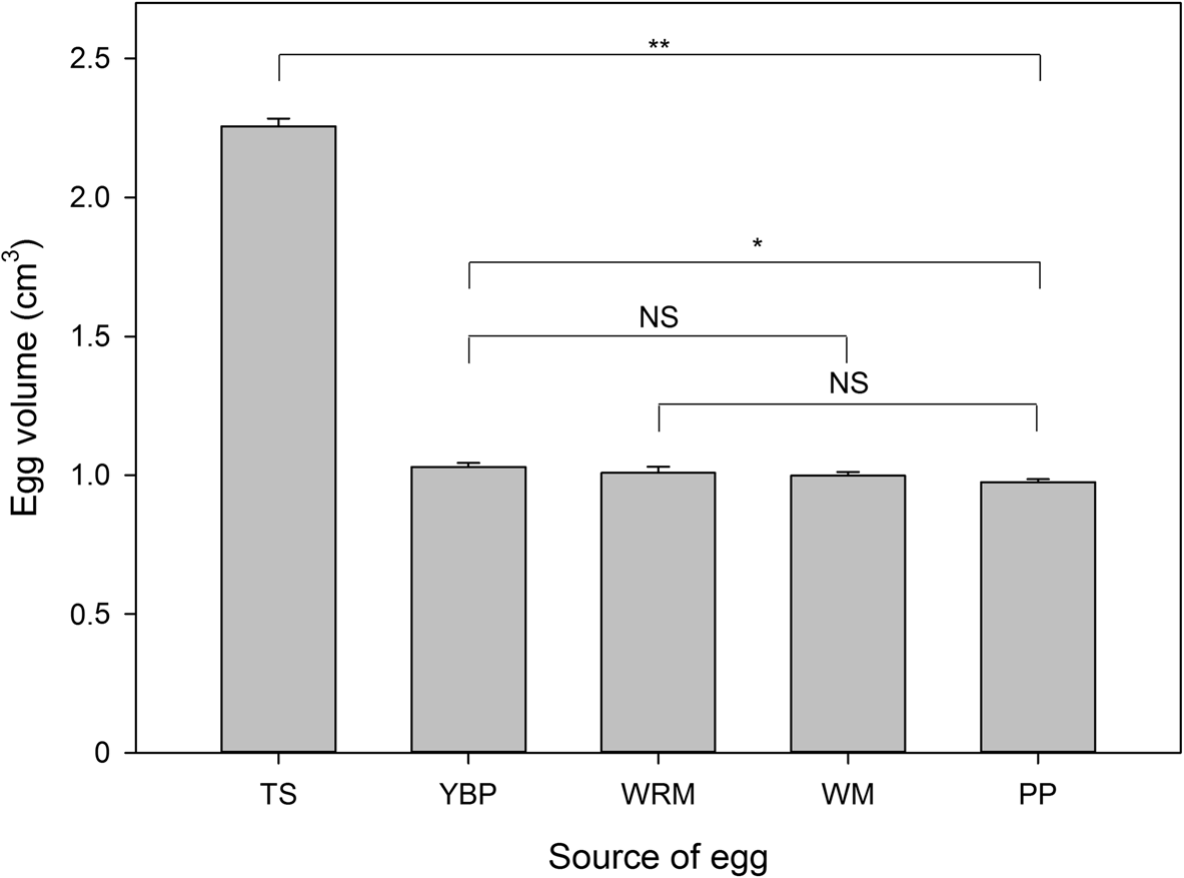
Sizes of parasitic eggs used in this study. (TS) tree sparrow; (YBP) yellow-bellied prinia; (WRM) white-rumped munia; (WM) white model; (PP) plain prinia. NS *P* ≥ 0.05; ^*^P < 0.05 and ^**^P < 0.01. *N* = 12 per group

A recent study showed that the rejection of non-mimetic model eggs by the Taiwanese plain prinia population was 4.8%, considerably lower than the rejection rate of the mainland population (63.6%) [11]. Based on these previous results using model eggs [11], we used various real eggs for our artificial parasitism experiments. Both island and mainland populations of plain prinias are grasp-type egg rejecters. However, the island population rejected only 4.76% of all white model eggs, compared to the 63.64% rejected by the mainland population [11]. In this study, the eggs of yellow-bellied prinia (*Prinia flaviventris*) were used as parasitic eggs for both mainland and island populations of the plain prinia. Plain prinia eggs (hereafter referred to as “host eggs”) are pale blue with scarlet markings (Fig. 1a), while yellow-bellied prinia eggs (hereafter referred to as “prinia eggs”) are covered with dense reddish markings (Fig. 1c). For the island population, tree sparrow (*Passer montanus*) and white-rumped munia (*Lonchura striata*) eggs were also used as parasitic eggs. Tree sparrow eggs are covered with dark brown markings (Fig. 1b), while munia eggs are immaculately white (Fig. 1d). The eggs used in our experiments were collected from the deserted nests of sympatric or nearly sympatric species. Eggs were kept in the dark in a cool insulated box until use. Egg volumes were calculated following Hoyt [17]. The sparrow eggs were significantly larger than all other parasitic eggs used, including the white model eggs used by Wang et al. (2016) [11] (ANOVA: F_4,55_ = 855.624, *P* < 0.001; least significant difference (LSD) test, pairwise comparison of sparrow eggs to all other eggs: *P* < 0.001 for all comparisons). Prinia eggs were slightly larger than host eggs (LSD test: *P* = 0.044), while the model eggs and the munia eggs were not significantly different in size from the host eggs (Fig. 2). However, the model eggs were solid and approximately 40% heavier than the host eggs (t = 15.723, df = 22, *P* < 0.001, Student’s *t* test).

### Artificial parasitism experiment

Mainland and island plain prinia nests were randomly allocated to one of three artificial parasitism trials: (1) the model egg trial, where one model egg was inserted into each host nest; (2) the real prinia egg trial, where one yellow-bellied prinia egg was inserted into each host nest; and (3) the conspecific trial, where one plain prinia egg from another nest was inserted into each host nest. An additional two trials were conducted using the island population: (4) the real munia egg trial; and (5) the real sparrow egg trail, where one munia or sparrow egg, respectively, was inserted into each host nest. The parasitic egg was inserted into the host nest on the day after the clutch completion or during the early stages of incubation (a floating test was used to estimate laying date) [18]. After insertion, the nest was monitored for 6 days to confirm parasitic egg fate. The parasitic egg was considered rejected if the egg was ejected or the nest was deserted, and was considered accepted otherwise (i.e., if the egg continued to be incubated) [11, 19].

### Statistical analyses

The likelihood ratio test was used to compare rejection rates, while the generalized linear model (GLM), with a binomial distribution and a logit link function, was used to assess the acceptance or rejection of the parasitic egg by the host with respect to the following variables: egg type (model or real), egg size [large (sparrow egg), medium (prinia egg), or small (munia or model egg)], interaction between egg type and population, interaction between egg size and population, clutch size, and egg laying date. One model included the independent variables egg type, interaction between egg type and population, clutch size, and egg laying date, while the other model included the independent variables egg size, interaction between egg size and population, clutch size, and egg laying date. Two models were used because egg type and egg size represented two different methods of classifying the same data. The omnibus test was used to compare the fitted model to the intercept-only model. All statistical analyses were performed using IBM SPSS 25.0 (IBM Inc., USA). All tests were two-tailed, and values were presented as means ± SE.

## Results

**Fig. 3:**
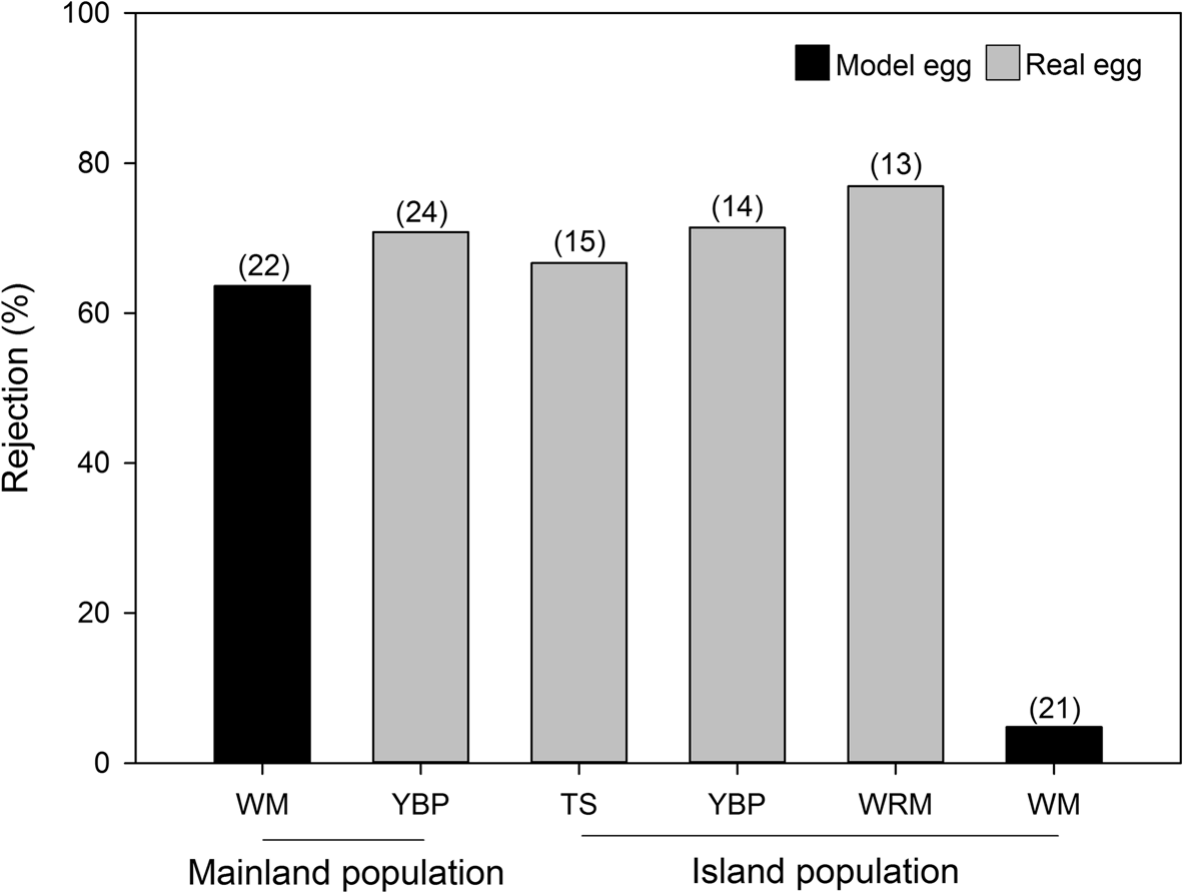
Rejection rates of various parasitic eggs by two populations of the plain prinia (*Prinia inornata*). Numbers on bars indicate sample sizes. (WM) white model; (YBP) yellow-bellied prinia; (TS) tree sparrow; and (WRM) white-rumped munia

Both the mainland and the Taiwanese plain prinia populations accepted all conspecific eggs (*n* = 12 for both populations), and consistently rejected prinia eggs: 70.83% were rejected by the mainland population, and 71.43% were rejected by the island population (likelihood ratio test: χ^2^ = 0.002, df = 1, *P* = 0.969). However, the island population accepted 95.24% of all white model eggs, significantly more than the mainland population (36.37%; likelihood ratio test: χ^2^ = 18.736, df = 1, *P* < 0.001; Fig. 3). Interestingly, the island population frequently rejected sparrow eggs (66.67%), prinia eggs (71.43%), and munia eggs (76.92%), even though these eggs differed in size (likelihood ratio test: χ^2^ = 0.362, df = 2, *P* = 0.834; Figs. 2 and 3). Furthermore, plain prinias rejected significantly more munia eggs than model eggs, even though these eggs were similar in color and size (likelihood ratio test: χ^2^ = 20.72, df = 1, *P* < 0.001). In addition, the GLM results indicated that only egg type and the interaction between egg type and population predicted host egg rejection (egg type: Wald = 12.121, df = 1, *P* < 0.001; egg type × population: Wald = 7.275, df = 2, *P* = 0.026; Table 1). Therefore, the mainland and island populations rejected real eggs at similar frequencies, but the island population rejected model eggs significantly less frequently than did the mainland population.

**Table 1:** Host responses to parasitic eggs, as assessed using a generalized linear model (GLM) with a binomial distribution and a logit link function

		χ^2ψ^	df	P
GLM for egg type	Omnibus test for model fit	34.32	5	< 0.001^**^
	Intercept	1.346	1	0.246
	egg type	12.12	1	< 0.001^**^
	egg type × population	7.275	2	0.026^*^
	laying date	0.024	1	0.877
	clutch size	1.386	1	0.239
GLM for egg size	Omnibus test for model fit	13.81	6	0.032^*^
	intercept	0.562	1	0.453
	egg size	5.689	2	0.058
	egg size × population	1.69	2	0.43
	laying date	0.028	1	0.867
	clutch size	1.23	1	0.267

^ψ^Likelihood ratio or Wald χ^2^ for Omnibus test or model effects test, respectively. ^*^P < 0.05; ^**^P < 0.01

## Discussion

The island and mainland populations rejected non-mimetic real eggs at similar rates. However, the rejection rate of non-mimetic model eggs by the Taiwanese plain prinia population was significantly lower after the release from brood parasitism. The GLM further showed that egg type (i.e., model or real) predicted host rejection behavior, and that mainland and island populations responded differently to different egg types. Model eggs are undoubtedly harder to eject, even for grasp rejecters like the plain prinia, because these eggs are solid and are heavier than real eggs. Soler et al. (2017) found that larger eggs are harder for grasp-type rejecters to eject, because the grasp-rejecter ejection ability depends on bill size [8]. In this study the model eggs were similar in size to the host eggs, but heavier. Thus, the island population of plain prinias rejected many fewer model eggs because these eggs were heavier and harder to grasp during ejection. That is, the heaver model eggs had a greater ejection cost, affecting the host decision to eject [10].

All parasitic eggs used in this study, with the exception of the conspecific eggs, were treated as non-mimetic eggs because they differed from host eggs in a variety of ways (Fig. 1). Although parasitic eggs with various phenotypes were used, our results were comparable for two reasons: first, model and prinia eggs were inserted into both mainland and island nests, and the mainland population rejected more model eggs than did the island population. Second, our comparison of the rejection rates of the similarly colored and -sized model and munia eggs indicated that the island population recognized white model eggs, but accepted them. In combination, these results suggested that differences in brood parasitism pressure may explain differences in model egg rejection frequency between mainland and island populations. For example, the island population might lack sufficient motivation for rejection, as is common in host populations with a low risk of parasitism [7]. That is, hosts under low parasitism pressure may accept parasite eggs more frequently (i.e., decide to reject less often), while hosts under high parasitism pressure may be more likely to decide to reject. Rejection motivation may be one of the important factors underlying this decision process. Recently, Soler et al. (2017) suggested that hosts with lower risks of parasitism might more frequently accept parasite eggs that are difficult to eject. The authors thus hypothesized that the absence of parasitism implies an absence of egg rejection stimuli; the host is not motivated to reject the foreign eggs because the motivation threshold is too high [8]. In addition, no obvious traces of pecking were detected on the accepted model eggs, suggesting that the absence of parasitism-associated stimuli might dramatically reduce egg rejection motivation in the island population.

Previous studies have shown that egg recognition should not be under directional selection, and thus can persist after the relaxation of parasitism [2, 20–23]. Indeed, host defense mechanisms were shown to persist for 280–300 million years as a result of evolutionary change, rather than phenotypic plasticity [22, 24]. Our results suggested that brood parasites are forced to become increasingly specialized once the host has evolved egg recognition capabilities, because these types of defenses are highly persistent [21].

In summary, our results suggested that the likelihood of model-egg rejection declined in the absence of brood parasitism, because some factors affecting the decision to reject, such as rejection motivation, varied with parasitism pressure. Moreover, we found that the island population rejected most of the real eggs, similar to the mainland population, but, in contrast to the mainland population, the island population accepted most of the model eggs. Therefore, the use of model eggs in tests of artificial parasitism might fail to detect egg recognition by non-parasitised hosts. This result has important implications for future studies of parasitism: model eggs should be used with caution, not only for small-billed host species [9], but also for host species that are grasp rejectors. In addition, care should be taken when using model eggs to compare egg-recognition abilities among populations within a single species, as well as among host species. Finally, model eggs may be inappropriate in comparative studies of host populations under different degrees of parasitic selection pressure. We recommend that such studies utilize real eggs.

## Data Availability

Data used in this study are available in the electronic supplementary material.
